# Supplementary material for: Spatial and temporal localization of immune transcripts defines hallmarks and diversity in the tuberculosis granuloma
Source: Nat Commun. 2019 Apr 23;10:1823. doi: 10.1038/s41467-019-09816-4 (PMC6479067; doi:10.1038/s41467-019-09816-4)
Supplement: Supplementary file 1 — Supplementary Information [file 41467_2019_9816_MOESM1_ESM.pdf]

Supplementary Information

Spatial and temporal localization of immune transcripts defines hallmarks and diversity in the tuberculosis granuloma

Carow et al.

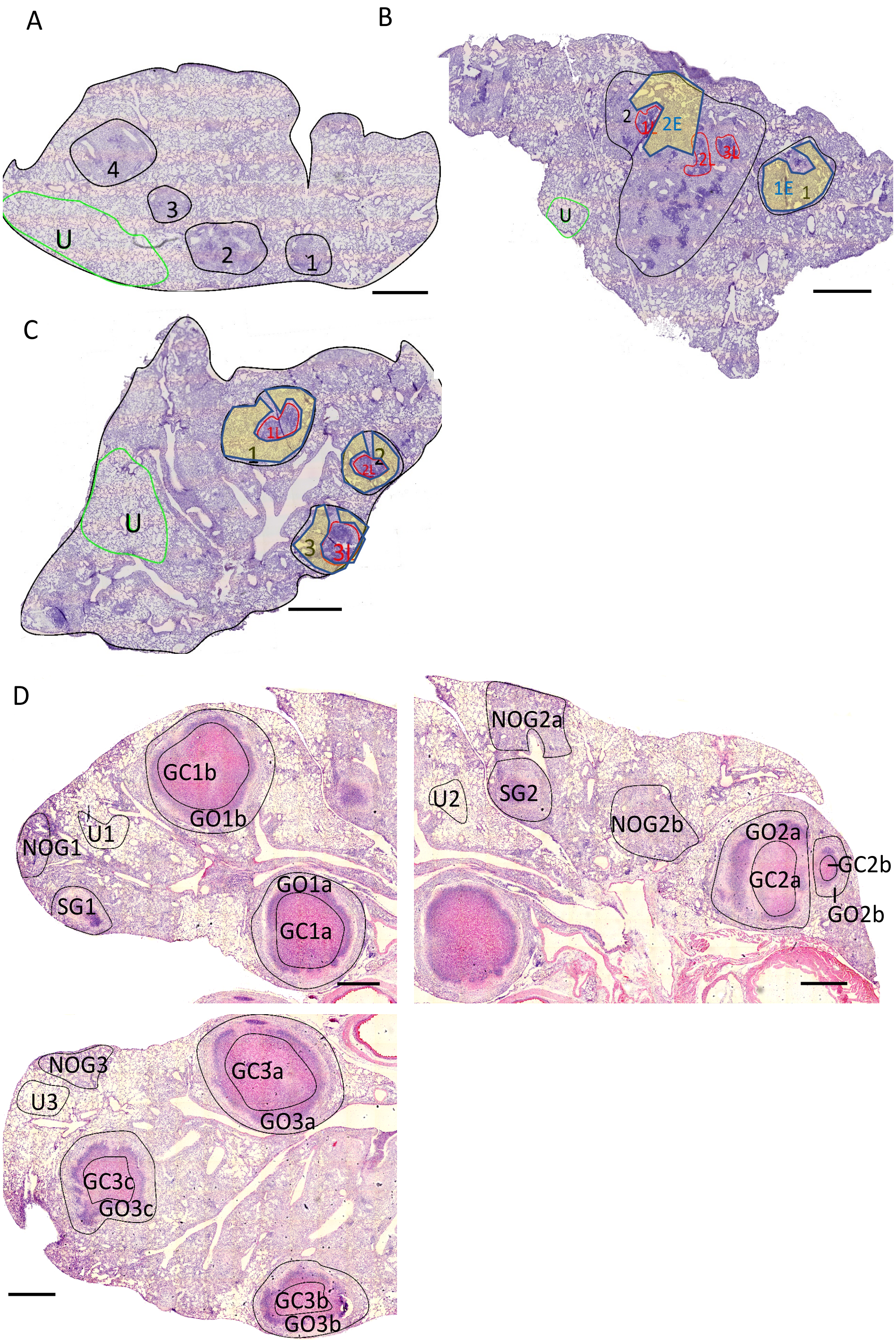

Supplementary Figure 1

Histological features of analyzed tissue sections

H&E stained sections from lung lobes from C57Bl6 mice at 3 (A), 8 (B), 12 (C) wpi. In D, a section from C3HeB/FeJ mice 10 weeks after infection are shown. Areas that are marked were found and analyzed thoughout the paper for all three consecutive sections. The areas occupied by lesions are marked and numbered in black. Unaffected

areas for all lungs are marked in green. In B and C, the lymphoid rich areas are marked in red and epitheloid areas shaded in yellow.

In D encapsulated granulomas and non-encapsulated lesions can be observed in three regions of a C3HeB/FeJ lung. U: unaffected area; GC: granuloma center; GO: granuloma outline; NOG: non-organized granuloma and SG: small necrotic granuloma. Scale bar: 1000 μm.

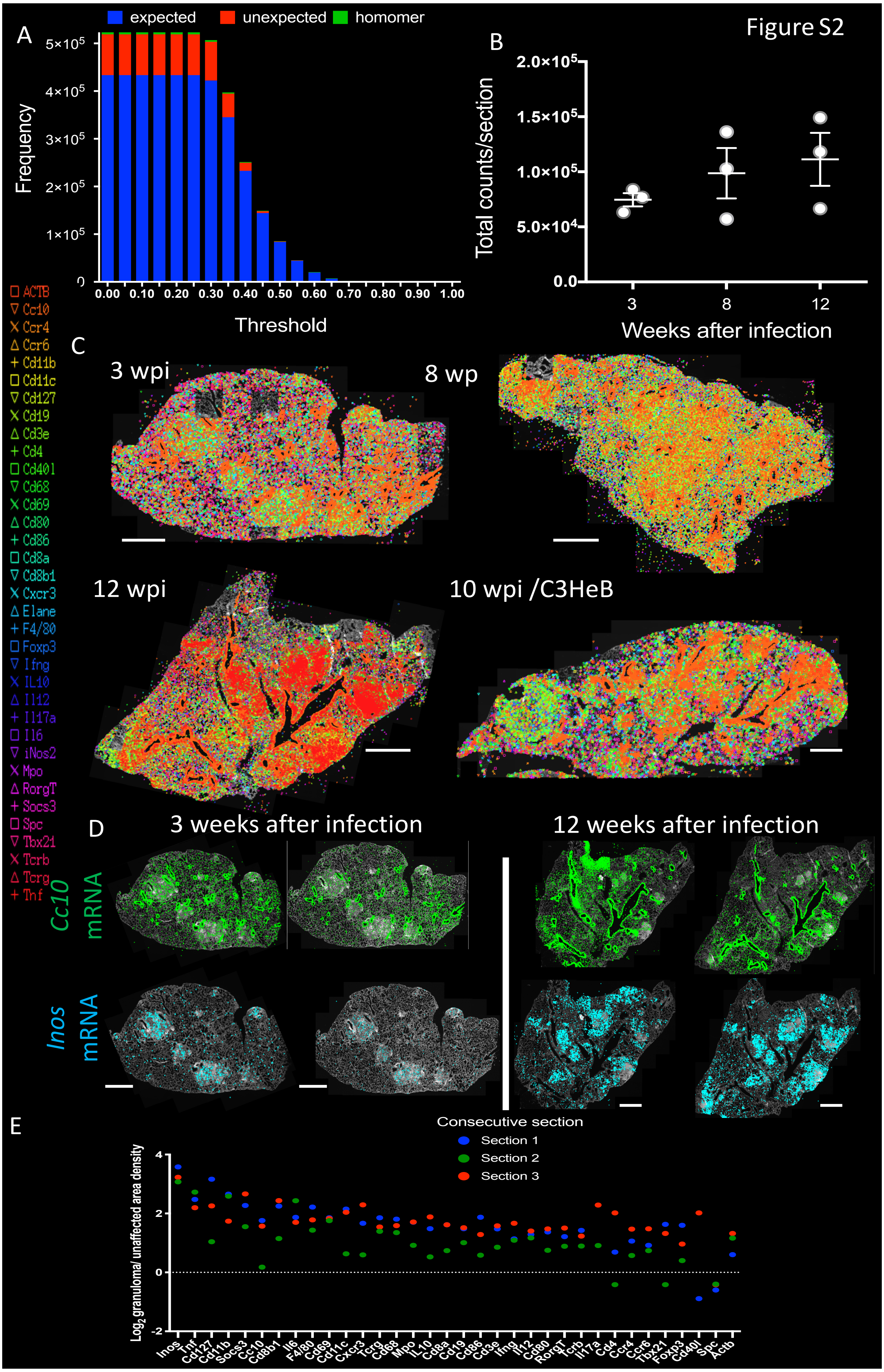
Figure S2
A
expected
unexpected
homomer
Frequency
Threshold
B
Total counts/section
Weeks after infection
C
3 wpi
8 wp
12 wpi
10 wpi /C3HeB
ACTB
Cc10
Ccr4
Ccr6
Cd11b
Cd11c
Cd127
Cd19
Cd3e
Cd4
Cd40l
Cd68
Cd69
Cd80
Cd86
Cd8a
Cd8b1
Cxcr3
Elane
F4/80
Foxp3
Ifng
IL10
Il12
Il17a
Il6
iNos2
Mpo
RorgT
Socs3
Spc
Tbx21
Tcrb
Tcrg
Tnf
D
3 weeks after infection
12 weeks after infection
Cc10 mRNA
Inos mRNA
E
Consecutive section
Section 1
Section 2
Section 3
Log2 granuloma/ unaffected area density

Supplementary Figure 2

Specificity and reproducibility of the in situ sequencing

A. The density of all barcoded sequences in relation to unexpected and homomers (unspecific) reads in each area at different signal intensity thresholds are depicted. The unexpected barcodes showed lower scores than true expected barcodes, and could thus be excluded from further analysis by setting a cut-off at a fixed threshold (0.45).

B. The mean ± SEM of the total number of amplified sequences per section in the different sections analysed are shown.

C. Raw data showing the location of all decoded sequences in paraformaldehyde-fixed lung sections from *M. tuberculosis* infected mice obtained at the indicated time points after infection. Each dot represents one decoded sequence. The sequences are aligned against a DAPI staining. Note the differential localization of sequences. Scale bar: 1000 μm.

D. Raw data showing the location of *Cc10* and *Inos* decoded transcripts called from two consecutive lung sections from a *M. tuberculosis*-infected mouse obtained at the indicated time points after infection. Each dot represents one decoded sequence. The sequences are aligned against a DAPI staining. Note the differential localization of both sequences and the similarity of consecutive images. Scale bar: 1000 μm

E. The relative fold increase in the density of individual sequences in granuloma vs unaffected lung areas from consecutive lung sections at 3 wpi are shown. A dotted line indicates the mean relative density of *Actb* mRNA. We have excluded from this graph transcripts showing either no signals in the unaffected areas or those in which more than 2 different areas showed no signals. Source data are provided as a Source Data file.

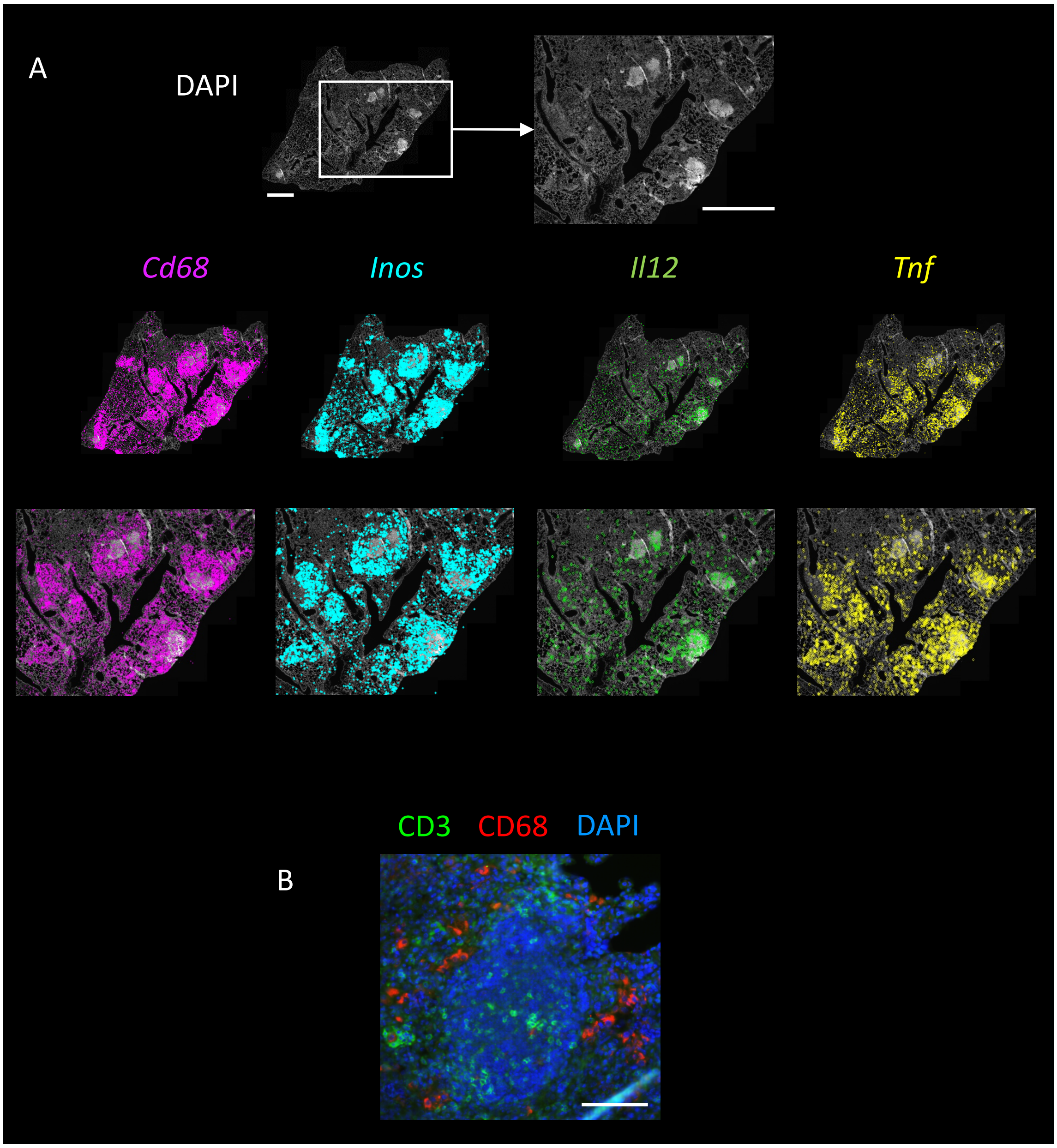

Supplementary Figure 3

Spatial distribution of myeloid transcripts in lungs from M. tuberculosis-infected mice

A. The raw signals for *Tnf, Inos, Il12* and *Cd68* (A) mRNAs in lungs from *M. tuberculosis* infected mice. Note in A that while *Inos* and *Cd68* sequences overlap, this is not the case for *Cd68* and *Il12* transcripts which locate in different areas of the granuloma, better shown in the zoomed area. Scale: 1000 µm

B. Double immunolabelling for CD3 and CD68 in DAPI stained C57BL/6 lung section 12 wpi with *M. tuberculosis*. A representative micrograph from 3 independent samples is shown. Scale: 100 µm

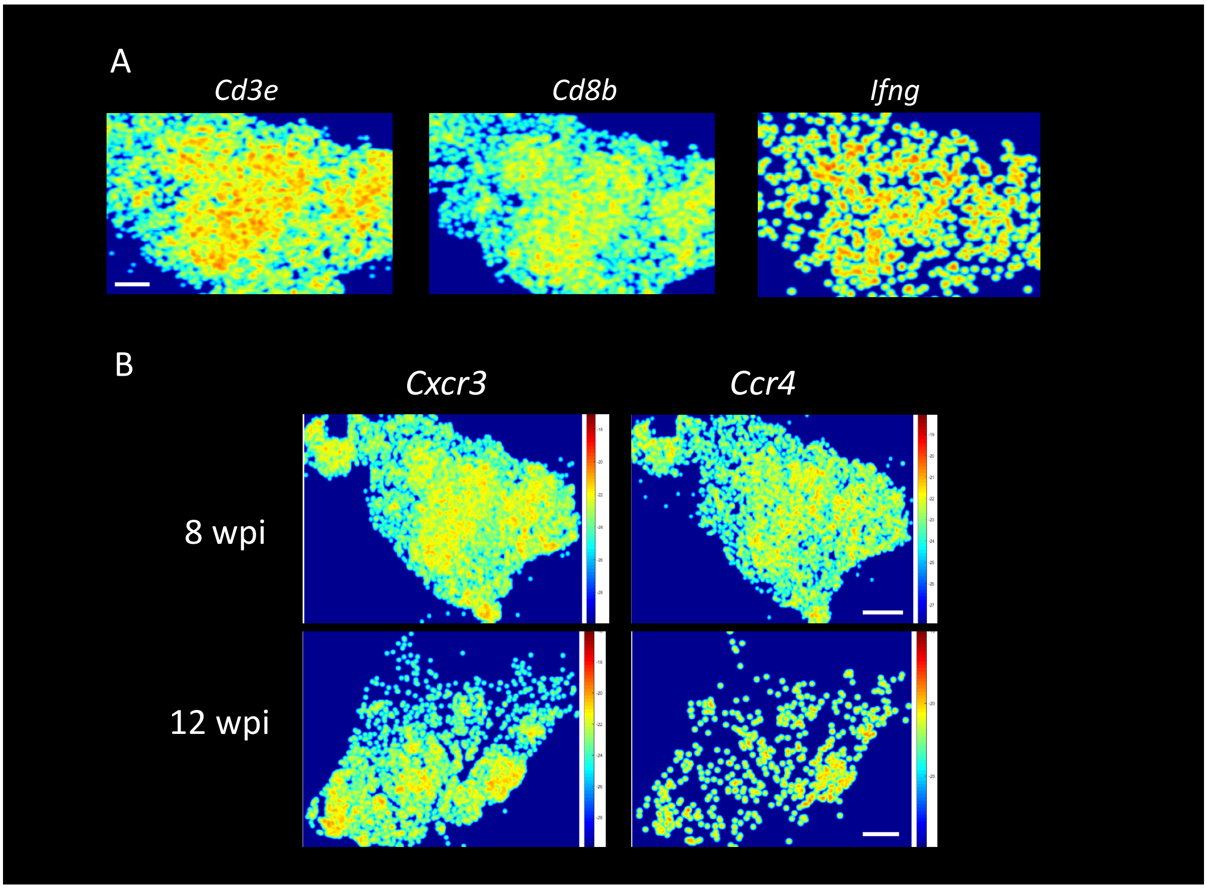

Supplementary Figure 4

Spatial distribution of lymphoid transcripts in lungs from M. tuberculosis-infected mice

A. Pseudocolor log2 density plots of *Cd3e, Cd8b* and *Ifng* mRNA for one representative lung at 8 wpi. Scale: 1000 µm

B. Density plot of *Cxcr3* and *Ccr4* sequences in lungs at 8 and 12 wpi. Scale bar: 1000 µm.

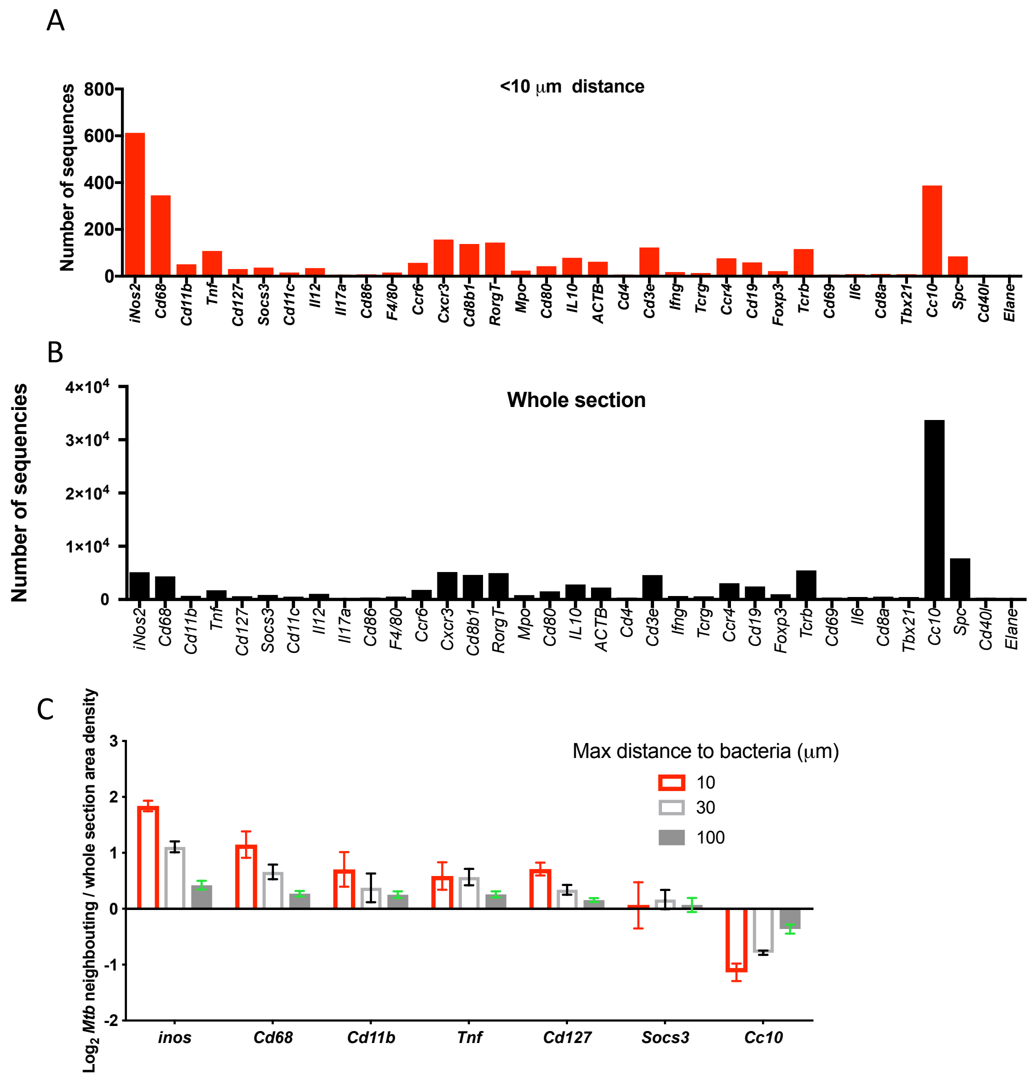

Supplementary Figure 5

Identification of sequences expressed at different distances from M. tuberculosis in the lung

A. The transcript counts for each sequence located at <10 µm from *M. tuberculosis*.

B. The transcript counts for each sequence in the whole area of the section in (A) are depicted.

C. The frequency of selected transcripts located at <10, 30 or 100 µm from *M. tuberculosis* bacteria in relation to the total sequence count for each distance was determined. The fold increase of such frequencies was compared to the

frequencies of each sequence in the whole section. The mean fold increase at each distance vs the whole section of 7 selected transcripts ± SEM of in 3 consecutive sections is depicted. Source data are provided as a Source Data file.

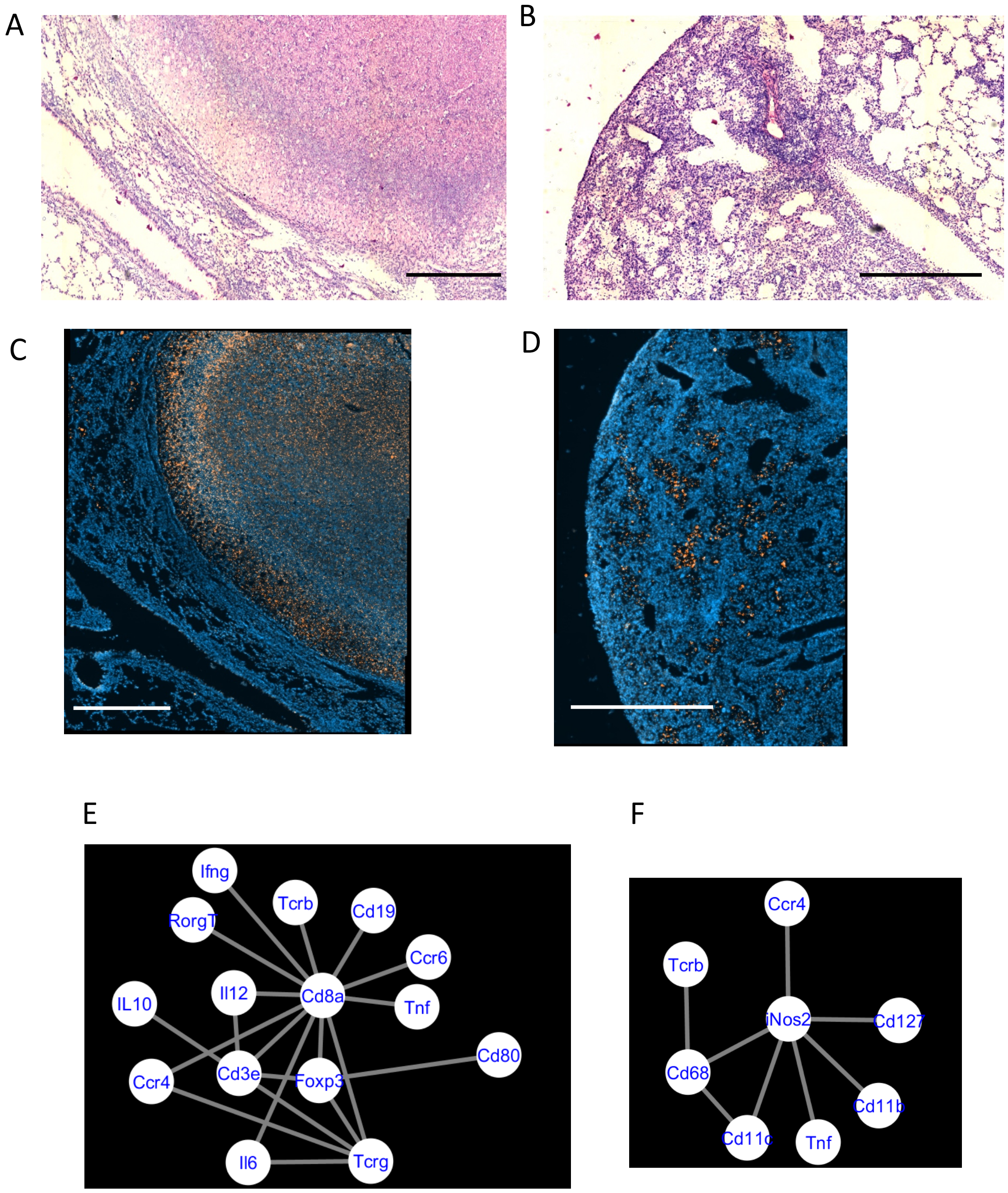

Supplementary Figure 6

In situ sequencing of the encapsulated granulomas

A-B. Micrograph showing the rim area from a necrotic encapsulated granuloma, showing the presence of a capsule, surrounding a layer of epitheloid and foamy cells and delineating compressed lung parenchyma and epitheloid and lymphoid cells (A). Area showing a perivascular lesion with numerous lymphocytes and epitheloid cells in the same H& E stained section from a C3HeB/FeJ lung (B). Scale bar: 500 µm.

C-D. Auramine-Rhodamine staining of C3HeB/FeJ encapsulated (C) and non-encapsulated lesions (D). Note bacteria localizes in the rim of the encapsulated lesion. Scale bar: 500 μm.

E-F. The networks of co-expressed transcripts in an encapsulated granuloma (E) and non-encapsulated cellular granuloma (F) are shown.

Supplementary tables

Supplementary Table 1

Counts and statistical analysis of paired sequence combinations

Sequences	R1L count-section 1	p value R1L section 1	R1L count section 2	p value R1L section 2
Ccr6-Cd19	105	0	302	0
Ccr6-Cd3e	61	0	126	0
Ccr6-Cd68	45	8,63E-05	99	2,36E-07
Ccr6-Cxcr3	16	5,46E-05	30	1,11E-16
Ccr6-IL10	9	1,89E-09	43	0
Ccr6-Il12	14	8,88E-16	22	0
Ccr6-RorgT	20	3,65E-08	56	5,77E-15
Cd19-Cd3e	130	0	323	0
Cd19-Cd68	81	3,26E-05	229	0
Cd19-Cd8b1	25	2,35E-12	37	0
Cd19-Cxcr3	31	1,34E-07	70	0
Cd19-Il12	18	5,74E-11	67	0
Cd19-RorgT	43	0	106	0
Cd19-Socs3	12	9,06E-08	21	0
Cd19-Tnf	27	3,84E-05	140	0
Cd3e-Cd68	100	4,33E-05	221	1,22E-15
Cd3e-Il12	25	5,55E-16	32	0
Cd68-iNos2	146	2,33E-09	286	0
Ifng-iNos2	16	3,07E-07	22	0

Example of the counts and p values of statistically significant sequence pairs (χ^2 test) at 10 μm in 1 lymphoid region (R1L see Supplementary Figure 1) of 2 consecutive lung tissue sections at 12 wpi are here shown.

Supplementary Table 2

cDNA primers

name	sequence
mSocs3_prim_2	CTGGAACTGCCCGGCCGGTC
mSocs3_prim_3_AP2	CTCTCTTGGGGGTACTCCCG
mSocs3_prim_4_AP3	TGGGCTCCAAGATGGCTCAT
mMpo_prim_2	GTGTTGGTTAAACTGAGTG
mMpo_prim_3	AGTTGAGGCCAGTGAAGAAG
mMpo_prim_4	GTGCCAACTCCAGGTTCTTC
mIl10_prim_2	AGTGCTGAGCCAGGCATGAT
mIl10_prim_3	TCAAATTCATTCATGGCCTT
mIl10_prim_4	AAGACCCATGAGTTTCTTCA
mIfng_prim_2	AGGAGGAGAAGCCCAGAACT
mIfng_prim_3	TCCGGCAACAGCTGGTGGAC
mIfng_prim_4	CTCTTGAGACACTGCTTTCT
mFoxp3_prim_2	CATATACCAGGCACAGTGCC
mFoxp3_prim_3	GACCAGGCCGGGAGCACACT
mFoxp3_prim_4	GGCATTGCTTGAGGCTGCGT
mCxcr3_prim_2	CAGGGCGGGGAGTCAGAGAA
mCxcr3_prim_3	GGGGTCCCTGCGGTAGATCT
mCxcr3_prim_4	TGGTCAGAGCGGCCCAGGCG
mCd80_prim_2	AAATGGAACAGAGTGTCTTT
mCd80_prim_3	ATATAAAGTCCGGTTCTTAT
mCd80_prim_4	GACGACGACTGTTATTACTG
mCd19_prim_2	CAGGAAGGGTGTTGACTGGT
mCd19_prim_3	CCCGAGGGAGGCGTCACTTT
mCd19_prim_4	CCCTTGTGGGACACCATGGA
mCcr6_prim_2	AGAACTGTGAAGTTGTTCAC
mCcr6_prim_3	ACTGCCACACAGATGACCTT
mCcr6_prim_4	AGATGTAGCTTTCCGAGTAA
mCcr4_prim_2	CTCCGGGTACCAGCAGGAGA
mCcr4_prim_3	GAAGAGTTGGGTGATGTACT
mCcr4_prim_4	ACTCCTGCCTCTGCCTCCAC
mCd11b_prim_2	GCCAGGTCCATCAAGCCATC

mCd11b_prim_3	CCAGCATCCTTGTTTTTTAA
mCd11b_prim_4	TGTTTCACATTTCTGCATCA
mCd3e_prim_2	CAGGATGCCCCAGAAAGTGT
mCd3e_prim_3	AGTCTGGGTTGGGAACAGGT
mCd3e_prim_4	AGGAGGTATGGGGTGTGTAA
mF4/80_prim_2	AAGAGGAGCAGCCAAAAGCC
mF4/80_prim_3	GAGCCTGGTACATTGGTGCA
mF4/80_prim_4	ACAGCAGGAAGGTGGCTATG
mCd4_prim_2	CTCGAGACTTTGCAAACAGG
mCd4_prim_3	GTATCTTGAGGGTGAGTGGG
mCd4_prim_4	TCTTGCAAATTCAAAAGAGA
mCd8b1_prim_2	CATCTTGGCGCTTTTGGGGA
mCd8b1_prim_3	CTGGGTCTCTGGGTGGGGGA
mCd8b1_prim_4	TCCAGGGTCCCGGCTAGCTC
mCd8a_prim_2	TGGGGGAGGCGTGTAGGGTC
mCd8a_prim_3	CTAGCTCTGGTGTTACAGTC
mCd8a_prim_4	CAGAAAGAGCCTGGGAATCT
mCd11c_prim_2	CCAGGTACAGCTCATGACTG
mCd11c_prim_3	CTGATTCTCAATATCCTTCA
mCd11c_prim_4	TAGTTGGGTCTTGGGGCTTT
mCd127_prim_2	TATAGCGAAAGCTCTACCCA
mCd127_prim_3	TGAATCTGGCAGTCCAGGAA
mCd127_prim_4	GACAGGTTCATGGCAAGAGA
mIl17a_prim_2	CAGATGAAGCTCTCCCTGGA
mIl17a_prim_3	TGGGGGTTTCTTAGGGGTCA
mIl17a_prim_4	AACATAAAACTAAGTTTGGT
miNos2_prim_2	ATCTCTCCACTGCCCCAGTT
miNos2_prim_3	TCCAGGATGTTGTAGCGCTG
miNos2_prim_4	GTCTAAAGGCTCCGGGCTCT
mTcrg_prim_2	GCAGGAAGTGCTCTGCAGGC
mTcrg_prim_3	ATGACATCGGGAAAGAACTT
mTcrg_prim_4	TTATGGCAGTGAGGATGAAA
mRorgT_prim_2	AGCTCCCGAGATGTCCGGTG
mRorgT_prim_3	ACTTCCTCTGGTAGCTGGTC
mRorgT_prim_4	TGTCAGTCTGTTTTTTATTT

mTcrb_prim_2	GGGTGCCTGCCGCAAAGTAG
mTcrb_prim_3	TCCAGGGTCCCGGCTAGCTC
mTcrb_prim_4	CTGGGTCTCTGGGTGGGGGA
mIl6_prim_3	TCGTTCTTGGTGGGCTCCAG
mIl6_prim_4	TTGTTCTTCATGTACTCCAG
mIl6_prim_5	ACTTATACATTCCAAGAAAC
mIl12_prim_3	GCAAGGGTGGCCAAAAAGAG
mIl12_prim_4	TTGTCTAGAATGATCTGCTG
mIl12_prim_5	GAGAGAAGCGATGGAGGGGA
mCd68_prim_3	TGCCTTCTCTTGGAAGAGGA
mCd68_prim_4	AGGATTCGGATTTGAATTTG
mCd68_prim_5	AGTGGACTGGGGCAGATGCT
mTnf_prim_4	TTTCTGTTCTCCCTCCTGGC
mTnf_prim_5	AAGAGAACCTGGGAGTAGAC
mTnf_prim_6	CATCTTGTGTTTCTGAGTAG
mSCGB1A1_prim	TATCTCTGAAATCCAGTGAG
mSftpc_prim	CAGGTCTCTCCCGGAAGAAT
Cd69_prim1	GTGCTTTTGTTTTCTTCCTT
Cd69_prim2	ATCCTTGATGTGATTAGCAG
Cd69_prim3	ATGTCTGATTAGCTTCATTT
Cd40lg_prim1	TCAGATTGTAAGTTCTTAGG
Cd40lg_prim2	CAGTCACGTTGACAAACACA
Cd40lg_prim3	CTTGAGTGTAGACATAATAG
mCD86_ap1_prim	CCAGATCTTAAGAGTCTGCA
mCD86_ap2_prim	CAAATATACCACTCCCATCC
mCD86_ap3_prim	CTGAAGTTGGCGATCACTGA
mElane_ap1_prim	CATTATGGCTTCGGATAATG
mElane_ap2_prim	TGGGCCACCTGCACGTTGGC
mElane_ap3_prim	GCCAGAGTCCGGCTGGATAG
mIl12b_ap1_prim	GAGTGTGGCCATTGTGTCCT
mIl12b_ap2_prim	GGTTGTCACAAAAGCTAATG
mIl12b_ap3_prim	GCACGTGAACCGTCCGGAGT
mTbx21_ap1_prim	AGTGATGCAAAACAGAAGAA
mTbx21_ap2_prim	CCCTGTTCCTCTGAGGATCC
mTbx21_ap3_prim	GTCGGGTCCTGTGCGCCCGG

mCd4_5_prim	CTTGAGGTCTTTGGTGGACT
mCd4_6_prim	AGAAGGAGATCCAGGGCTGG
mCd4_7_prim	GAACCCTTAGGTTGGACATG

Supplementary Table 3

Padlock probes (all padlock probes are 5' phosphorylated)

name	sequence
mSocs3_GGAG	TCCAACGTGGCCACCCTCTTCCTCTATGATTACTGACTGCGTCT ATTTAGTGGAGCCGGAGCTATCTTCTTTTACTGAGCCGACCTCTCTCC
mMpo_GAGG	CGATGACCCCTGCCTCCTCTTCCTCTATGATTACTGACTGCGTCT ATTTAGTGGAGCCGAGGCTATCTTCTTTTGCCCTTTGACAGCCTGCA
mIl10_CCGG	GCTAACCGACTCCTTAATGCTTCCTCTATGATTACTGACTGCGT CTATTTAGTGGAGCCCCGGCTATCTTCTTTGACCAGCTGGACAACATAC T
mIfng_CACA	GCTGTTTCTGGCTGTTACTGTTCCTCTATGATTACTGACTGCGTC TATTTAGTGGAGCCCACACTATCTTCTTTCTTTGCAGCTCTTCCTCATG
mFoxp3_CAAC	CCTCCCACCACCTTCTGCTTTCCTCTATGATTACTGACTGCGTCT ATTTAGTGGAGCCCAACCTATCTTCTTTGACAACCCAGCCATGATCAG
mCxcr3_AGAA	GCCCTCTACAGCCTCCTCTTTTCCTCTATGATTACTGACTGCGTC TATTTAGTGGAGCCAGAACTATCTTCTTTTTGACAGAACCTTCCTGCCA
mCd80_ACCA	CCGGGGCACATACAGCTGTTTCCTCTATGATTACTGACTGCGTC TATTTAGTGGAGCCACCACTATCTTCTTTCTGGGCCTGGTCCTTTCAGA
mCd19_ACAC	GGACTCCTCACCTGTCTCTTTTCCTCTATGATTACTGACTGCGTC TATTTAGTGGAGCCACACCTATCTTCTTTTGTGCTGCCATGCCTCCC
mCcr6_AAGA	GTTACTCATGCCACCAACACTTCCTCTATGATTACTGACTGCGT CTATTTAGTGGAGCCAAGACTATCTTCTTTCACCCTACCGTTCTGGGCA
mCcr4_AACC	TTTGCTGTTCGTCCTGTCCCTTCCTCTATGATTACTGACTGCGTC TATTTAGTGGAGCCAACCCTATCTTCTTTCTGAACCTGGCCATCTCGGA
mCd11b_AATT	AAACCCTAGCCCAAGATTCCTCTATGATTACTGACTGCGTCTAT TTAGTGGAGCCAATTCTATCTTCTTTACCTTCAATGACTTCAAGAG
mCd3e_ACGT	CACCCTGCTACTCCTTTCCTCTATGATTACTGACTGCGTCTATTT AGTGGAGCCACGTCTATCTTCTTTATCCAGCCCTCCGAG
mF4/80_ACTG	TGTCTGCTCAACCGTTCCTCTATGATTACTGACTGCGTCTATTTA GTGGAGCCACTGCTATCTTCTTTCTTTCATCTTCCTCATTCAC
mCd4_AGTC	TGCACCGTGACCCTGTCCTCTATGATTACTGACTGCGTCTATTTA GTGGAGCCAGTCCTATCTTCTTTCAGCGACTTCTGGAAC
mCd8b1_ATAT	GTTCAAACCAACCATACGTCCTCTATGATTACTGACTGCGTCTA TTTAGTGGAGCCATATCTATCTTCTTTCCTTCGTCCCTGCTG
mCd8a_ATCG	AAGTGAACTCTACTACTACCTCCTCTATGATTACTGACTGCGTC TATTTAGTGGAGCCATCGCTATCTTCTTTGTGCCAGTCCTTCAGA
mCd11c_ATGC	CTGCCACCAACCCTTTCCTCTATGATTACTGACTGCGTCTATTTA GTGGAGCCATGCCTATCTTCTTTGCCTGTCCCTTGCTG
mCd127_ATTA	CTTCTCTATTCTTTCTCTCTTCCTCTATGATTACTGACTGCGTCTA TTTAGTGGAGCCATTACTATCTTCTTTCACATACAAGCGTGCTT
mIl17a_CATG	AGGCAGCCTAAACAGATCCTCTATGATTACTGACTGCGTCTATT

	TAGTGGAGCCCATGCTATCTTCTTTCCTCGATTGTCCGCC
miNos2_GACT	AAGGCCACATCGGATTTCCTCTATGATTACTGACTGCGTCTATTTAGTGGAGCCGACTCTATCTTCTTTCATGACACTCTTCACCAC
mTcrg_GCCG	GACCTACCTTTGTCTCCTTCCTCTATGATTACTGACTGCGTCTATTTAGTGGAGCCGCCGCTATCTTCTTTAAATCTCCATAAGGCTGG
mRorgT_GCGC	TCCCTTTCTGCACTCTATTCCTCTATGATTACTGACTGCGTCTATTTAGTGGAGCCGCGCCTATCTTCTTTCCTTCACCCAGCCTT
mTcrb_GTAC	GTCTTGTCTGCCACCATCCTCTATGATTACTGACTGCGTCTATTTAGTGGAGCCGTACCTATCTTCTTTCACATCCTATCAACAAGGG
mActb_GCAT	TTACACCCTTTCTTTGACAATCCGAGTAGTCTTTGTGCGTCTATTTAGTGGAGCCGCATCTATCTTCTTTGACTGTTACTGAGCTGCGTT
mIl6_CGAT_1	CTCTACGAAGAACTGACAATTCCTCTATGATTACTGACTGCGTCTATTTAGTGGAGCCCGATCTATCTTCTTTGGTATCTGACTTATGTTGTT
mIl6_CGAT_2	GCTCTCCTAACAGATAAGCTTCCTCTATGATTACTGACTGCGTCTATTTAGTGGAGCCCGATCTATCTTCTTTTTCCTACCCCAATTTCCAAT
mIl12_CAGT_1	TGACACCTTTGCTGATTTCTTCCTCTATGATTACTGACTGCGTCTATTTAGTGGAGCCCAGTCTATCTTCTTTCAGTTTTCCTAAGTTCATCA
mIl12_CAGT_2	TCTCCCTCAAGTTCTTTGTTTCCTCTATGATTACTGACTGCGTCTATTTAGTGGAGCCCAGTCTATCTTCTTTGCACTCCCCATTCCTACT
mCd68_AGCT_1	CTGTCTCTCTCATTTCCTTATCCTCTATGATTACTGACTGCGTCTATTTAGTGGAGCCAGCTCTATCTTCTTTGACGGTACCCATCCCCAC
mCd68_AGCT_2	TCCTTCACGATGACACCTTCCTCTATGATTACTGACTGCGTCTATTTAGTGGAGCCAGCTCTATCTTCTTTGCCGTTACTCTCCTGCCA
mTnf_CCAA_3	ATGGCCCAGACCCTCACACTTCCTCTATGATTACTGACTGCGTCTATTTAGTGGAGCCCCAACTATCTTCTTTGCCTCCCTCTCATCAGTTCT
mSocs3_GGAG_2_AP1	GACCGGCCGGGCAGTTCCAGTCTACGATTTTACCAGTGGCTTTTGCGTCTATTTAGTGGAGCCGGAGCTATCTTCTTTGTGACTAAACATTACAAGAA
mSocs3_GGAG_3_AP2	CGGGAGTACCCCCAAGAGAGCTGATTCCTTTGACTCACATTTTTGCGTCTATTTAGTGGAGCCGGAGCTATCTTCTTTCCACCTGCCCAGGCACTCCC
mSocs3_GGAG_4_AP3	ATGAGCCATCTTGGAGCCCATCTACGAGTTTGCAGTCACGTTTTGCGTCTATTTAGTGGAGCCGGAGCTATCTTCTTTGAAGGGAGGCAGATCAACAG
mMpo_GAGG_2_AP1	CACTCAGTTTAACCAACACTCTACGATTTTACCAGTGGCTTTTGCGTCTATTTAGTGGAGCCGAGGCTATCTTCTTTGTTCTTTCAAAGGATTTGGG
mMpo_GAGG_3_AP2	CTTCTTCACTGGCCTCAACTCTGATTCCTTTGACTCACATTTTTGCGTCTATTTAGTGGAGCCGAGGCTATCTTCTTTGAGCCAGCTACCCGGTTCTC
mMpo_GAGG_4_AP3	GAAGAACCTGGAGTTGGCACTCTACGAGTTTGCAGTCACGTTTTGCGTCTATTTAGTGGAGCCGAGGCTATCTTCTTTGGTGAGCTCGGCACGGTGCT
mIl10_CCGG_2_AP1	ATCATGCCTGGCTCAGCACTTCTACGATTTTACCAGTGGCTTTTGCGTCTATTTAGTGGAGCCCCGGCTATCTTCTTTCTTGCAGAAAAGAGAGCTCC
mIl10_CCGG_3_AP2	AAGGCCATGAATGAATTTGACTGATTCCTTTGACTCACATTTTTGCGTCTATTTAGTGGAGCCCCGGCTATCTTCTTTTCCAAGACCAAGGTGTCTAC
mIl10_CCGG_4_AP3	TGAAGAAACTCATGGGTCTTTCTACGAGTTTGCAGTCACGTTTTGCGTCTATTTAGTGGAGCCCCGGCTATCTTCTTTCAGAGCTCCTAAGAG

	AGTTG
mIfng_CACA_2_AP1	AGTTCTGGGCTTCTCCTCCTTCTACGATTTACCAGTGGCTTTTGCGTCTATTTAGTGGAGCCCACACTATCTTCTTTACCCTCTGACTTGAGACAGA
mIfng_CACA_3_AP2	GTCCACCAGCTGTTGCCGGACTGATTCCTTTGACTCACATTTTTGCGTCTATTTAGTGGAGCCCACACTATCTTCTTTTCAATGAGCTCATCCGAGTG
mIfng_CACA_4_AP3	AGAAAGCAGTGTCTCAAGAGTCTACGAGTTTGCAGTCACGTTTTGCGTCTATTTAGTGGAGCCCACACTATCTTCTTTAGTAACAGGCTGTCCCTGAA
mFoxp3_CAAC_2_AP1	GGCACTGTGCCTGGTATATGTCTACGATTTACCAGTGGCTTTTGCGTCTATTTAGTGGAGCCCAACCTATCTTCTTTCTCTGCAGGTTTAGTGCTGT
mFoxp3_CAAC_3_AP2	AGTGTGCTCCCGGCCTGGTCCTGATTCCTTTGACTCACATTTTTGCGTCTATTTAGTGGAGCCCAACCTATCTTCTTTTAGCCACCAGTACTCAGGGC
mFoxp3_CAAC_4_AP3	ACGCAGCCTCAAGCAATGCCTCTACGAGTTTGCAGTCACGTTTTGCGTCTATTTAGTGGAGCCCAACCTATCTTCTTTACACAGGCATAACTGATCAT
mCxcr3_AGAA_2_AP1	TTCTCTGACTCCCCGCCCTGTCTACGATTTACCAGTGGCTTTTGCGTCTATTTAGTGGAGCCAGAACTATCTTCTTTATGGGGAAAACGAGAGCGAC
mCxcr3_AGAA_3_AP2	AGATCTACCGCAGGGACCCCCTGATTCCTTTGACTCACATTTTTGCGTCTATTTAGTGGAGCCAGAACTATCTTCTTTGAGCATAGTGCACGCCACCC
mCxcr3_AGAA_4_AP3	CGCCTGGGCCGCTCTGACCATCTACGAGTTTGCAGTCACGTTTTGCGTCTATTTAGTGGAGCCAGAACTATCTTCTTTAAATGTGGATGTTGTTCACG
mCd80_ACCA_2_AP1	AAAGACACTCTGTTCCATTTTCTACGATTTACCAGTGGCTTTTGCGTCTATTTAGTGGAGCCACCACTATCTTCTTTGGTTGTGAAACTCAACCTTC
mCd80_ACCA_3_AP2	ATAAGAACCGGACTTTATATCTGATTCCTTTGACTCACATTTTTGCGTCTATTTAGTGGAGCCACCACTATCTTCTTTACTAAAAGTGTGGCCCGAGT
mCd80_ACCA_4_AP3	CAGTAATAACAGTCGTCGTCTCTACGAGTTTGCAGTCACGTTTTGCGTCTATTTAGTGGAGCCACCACTATCTTCTTTCTTTGGGGCAGGATTCGGCG
mCd19_ACAC_2_AP1	ACCAGTCAACACCCTTCCTGTCTACGATTTACCAGTGGCTTTTGCGTCTATTTAGTGGAGCCACACCTATCTTCTTTGCTGGCTTGGTATCGAGGTA
mCd19_ACAC_3_AP2	AAAGTGACGCCTCCCTCGGGCTGATTCCTTTGACTCACATTTTTGCGTCTATTTAGTGGAGCCACACCTATCTTCTTTACCCCGCCAGGAGATTCTTC
mCd19_ACAC_4_AP3	TCCATGGTGTCCCACAAGGGTCTACGAGTTTGCAGTCACGTTTTGCGTCTATTTAGTGGAGCCACACCTATCTTCTTTAATATGTCCAGCCGCTACAT
mCcr6_AAGA_2_AP1	GTGAACAACTTCACAGTTCTTCTACGATTTACCAGTGGCTTTTGCGTCTATTTAGTGGAGCCAAGACTATCTTCTTTTGCTTCCTGGCCACCGAGGT
mCcr6_AAGA_3_AP2	AAGGTCATCTGTGTGGCAGTCTGATTCCTTTGACTCACATTTTTGCGTCTATTTAGTGGAGCCAAGACTATCTTCTTTCCAGAACACTGACGCACAGT

mCcr6_AAGA_4_AP3	TTACTCGGAAAGCTACATCTTCTACGAGTTTGCAGTCACGTTTTGCGTCTATTTAGTGGAGCCAAGACTATCTTCTTTGGCTTCCTCTGTGCCCGGGT
mCcr4_AACC_2_AP1	TCTCCTGCTGGTACCCGGAGTCTACGATTTTACCAGTGGCTTTTGCGTCTATTTAGTGGAGCCAACCCTATCTTCTTTCTCAACTGTTCTCATTGGCT
mCcr4_AACC_3_AP2	AGTACATCACCCAACTCTTCCTGATTCCTTTGACTCACATTTTTGCGTCTATTTAGTGGAGCCAACCCTATCTTCTTTTCTCGGGGAGAAATTCCGCA
mCcr4_AACC_4_AP3	GTGGAGGCAGAGGCAGGAGTTCTACGAGTTTGCAGTCACGTTTTGCGTCTATTTAGTGGAGCCAACCCTATCTTCTTTGTTATTGGGTATTGGGCATG
mCd11b_AATT_2_AP1	GATGGCTTGATGGACCTGGCTCTACGATTTTACCAGTGGCTTTTGCGTCTATTTAGTGGAGCCAATTCTATCTTCTTTGGGGTAAGGATCTCACAATG
mCd11b_AATT_3_AP2	TTAAAAAACAAGGATGCTGGCTGATTCCTTTGACTCACATTTTTGCGTCTATTTAGTGGAGCCAATTCTATCTTCTTTTTGCATGTCAAGAACAAGTA
mCd11b_AATT_4_AP3	TGATGCAGAAATGTGAAACATCTACGAGTTTGCAGTCACGTTTTGCGTCTATTTAGTGGAGCCAATTCTATCTTCTTTGCGCACCCAGGTCTTTGGAT
mCd3e_ACGT_2_AP1	ACACTTTCTGGGGCATCCTGTCTACGATTTTACCAGTGGCTTTTGCGTCTATTTAGTGGAGCCACGTCTATCTTCTTTTTCTGAGAGGATGCGGTGGA
mCd3e_ACGT_3_AP2	ACCTGTTCCCAACCCAGACTCTGATTCCTTTGACTCACATTTTTGCGTCTATTTAGTGGAGCCACGTCTATCTTCTTTCAAAACAAGGAGCGGCCACC
mCd3e_ACGT_4_AP3	TTACACACCCCATACCTCCTTCTACGAGTTTGCAGTCACGTTTTGCGTCTATTTAGTGGAGCCACGTCTATCTTCTTTCCCGGCCCATCCTCCGACTG
mF4/80_ACTG_2_AP1	GGCTTTTGGCTGCTCCTCTTTCTACGATTTTACCAGTGGCTTTTGCGTCTATTTAGTGGAGCCACTGCTATCTTCTTTACTGCCACAGTACGATGTGG
mF4/80_ACTG_3_AP2	TGCACCAATGTACCAGGCTCCTGATTCCTTTGACTCACATTTTTGCGTCTATTTAGTGGAGCCACTGCTATCTTCTTTAATGTGGACTGAATTCTGTC
mF4/80_ACTG_4_AP3	CATAGCCACCTTCCTGCTGTTCTACGAGTTTGCAGTCACGTTTTGCGTCTATTTAGTGGAGCCACTGCTATCTTCTTTCTGGTATGTCTTGCCTTGGC
mCd4_AGTC_2_AP1	CCTGTTTGCAAAGTCTCGAGTCTACGATTTTACCAGTGGCTTTTGCGTCTATTTAGTGGAGCCAGTCCTATCTTCTTTACTCTCTTCTTCACTAGGTA
mCd4_AGTC_3_AP2	CCCACTCACCCTCAAGATACCTGATTCCTTTGACTCACATTTTTGCGTCTATTTAGTGGAGCCAGTCCTATCTTCTTTCTCCAGCTGAAGGAAACGCT
mCd4_AGTC_4_AP3	TCTCTTTTGAATTTGCAAGATCTACGAGTTTGCAGTCACGTTTTGCGTCTATTTAGTGGAGCCAGTCCTATCTTCTTTAGCTTCCCATGATGCCTGCT
mCd8b1_ATAT_2_AP1	TCCCCAAAAGCGCCAAGATGTCTACGATTTTACCAGTGGCTTTTGCGTCTATTTAGTGGAGCCATATCTATCTTCTTTGAGCACTGAGGGGAACAGTG
mCd8b1_ATAT_3_AP2	TCCCCCACCCAGAGACCCAGCTGATTCCTTTGACTCACATTTTTGCGTCTATTTAGTGGAGCCATATCTATCTTCTTTGAAGAAGAAGCAATG

	CCCGT
mCd8b1_ATAT_4_AP3	GAGCTAGCCGGGACCCTGGATCTACGAGTTTGCAGTCACGTTTTGCGTCTATTTAGTGGAGCCATATCTATCTTCTTTCTTTGGCCTTTCATGGAAAA
mCd8a_ATCG_2_AP1	GACCCTACACGCCTCCCCCATCTACGATTTTACCAGTGGCTTTTGCGTCTATTTAGTGGAGCCATCGCTATCTTCTTTGCTAAAGGAGCAGTTTCCCC
mCd8a_ATCG_3_AP2	GACTGTAACACCAGAGCTAGCTGATTCCTTTGACTCACATTTTTGCGTCTATTTAGTGGAGCCATCGCTATCTTCTTTTACAATGGGAGTAATGAGCA
mCd8a_ATCG_4_AP3	AGATTCCCAGGCTCTTTCTGTCTACGAGTTTGCAGTCACGTTTTGCGTCTATTTAGTGGAGCCATCGCTATCTTCTTTTAAAGATTTTTATCTCAGTG
mCd11c_ATGC_2_AP1	CAGTCATGAGCTGTACCTGGTCTACGATTTTACCAGTGGCTTTTGCGTCTATTTAGTGGAGCCATGCCTATCTTCTTTCAGAGCCTGCTTCTGTTCTC
mCd11c_ATGC_3_AP2	TGAAGGATATTGAGAATCAGCTGATTCCTTTGACTCACATTTTTGCGTCTATTTAGTGGAGCCATGCCTATCTTCTTTCGTGGAGAACTTTGATGCTT
mCd11c_ATGC_4_AP3	AAAGCCCCAAGACCCAACTATCTACGAGTTTGCAGTCACGTTTTGCGTCTATTTAGTGGAGCCATGCCTATCTTCTTTTGTCTGCCTTCATATTCATG
mCd127_ATTA_2_AP1	TGGGTAGAGCTTTCGCTATATCTACGATTTTACCAGTGGCTTTTGCGTCTATTTAGTGGAGCCATTACTATCTTCTTTCTCTCTCAGAATGATGGCTC
mCd127_ATTA_3_AP2	TTCCTGGACTGCCAGATTCACTGATTCCTTTGACTCACATTTTTGCGTCTATTTAGTGGAGCCATTACTATCTTCTTTTGAGTTTCAATCCCGAAAGT
mCd127_ATTA_4_AP3	TCTCTTGCCATGAACCTGTCTCTACGAGTTTGCAGTCACGTTTTGCGTCTATTTAGTGGAGCCATTACTATCTTCTTTACACATTCCAACTTCAACCC
mIl17a_CATG_2_AP1	TCCAGGGAGAGCTTCATCTGTCTACGATTTTACCAGTGGCTTTTGCGTCTATTTAGTGGAGCCCATGCTATCTTCTTTTCAGGACGCGCAAACATGAG
mIl17a_CATG_3_AP2	TGACCCCTAAGAAACCCCCACTGATTCCTTTGACTCACATTTTTGCGTCTATTTAGTGGAGCCCATGCTATCTTCTTTCCTAAACAGAGACCCGCGGC
mIl17a_CATG_4_AP3	ACCAAACTTAGTTTTATGTTTCTACGAGTTTGCAGTCACGTTTTGCGTCTATTTAGTGGAGCCCATGCTATCTTCTTTCTCCTCTGAATGGGGTGAAA
miNos2_GACT_2_AP1	AACTGGGGCAGTGGAGAGATTCTACGATTTTACCAGTGGCTTTTGCGTCTATTTAGTGGAGCCGACTCTATCTTCTTTCACAGTATGTGAGGATCAAA
miNos2_GACT_3_AP2	CAGCGCTACAACATCCTGGACTGATTCCTTTGACTCACATTTTTGCGTCTATTTAGTGGAGCCGACTCTATCTTCTTTTTCGAGACTTCTGTGACACA
miNos2_GACT_4_AP3	AGAGCCCGGAGCCTTTAGACTCTACGAGTTTGCAGTCACGTTTTGCGTCTATTTAGTGGAGCCGACTCTATCTTCTTTACAGCAATATAGGCTCATCC
mTcrg_GCCG_2_AP1	GCCTGCAGAGCACTTCCTGCTCTACGATTTTACCAGTGGCTTTTGCGTCTATTTAGTGGAGCCGCCGCTATCTTCTTTGCTGGTGACCTGAAATTCCA

mTcrg_GCCG_3_AP2	AAGTTCTTTCCCGATGTCATCTGATTCCTTTGACTCACATTTTTGCGTCTATTTAGTGGAGCCGCCGCTATCTTCTTTCATACCTTTGTCTCCTTGAA
mTcrg_GCCG_4_AP3	TTTCATCCTCACTGCCATAATCTACGAGTTTGCAGTCACGTTTTGCGTCTATTTAGTGGAGCCGCCGCTATCTTCTTTGGTACAGCAAGTCAGCTGGA
mRorgT_GCGC_2_AP1	CACCGGACATCTCGGGAGCTTCTACGATTTTACCAGTGGCTTTTGCGTCTATTTAGTGGAGCCGCGCCTATCTTCTTTACAGGGCCCCACAGAGACAC
mRorgT_GCGC_3_AP2	GACCAGCTACCAGAGGAAGTCTGATTCCTTTGACTCACATTTTTGCGTCTATTTAGTGGAGCCGCGCCTATCTTCTTTCTCTTTTCACGGGAGGAGGT
mRorgT_GCGC_4_AP3	AAATAAAAAACAGACTGACATCTACGAGTTTGCAGTCACGTTTTGCGTCTATTTAGTGGAGCCGCGCCTATCTTCTTTAGAGATAGGATGACCAAGTC
mTcrb_GTAC_2_AP1	CTACTTTGCGGCAGGCACCCTCTACGATTTTACCAGTGGCTTTTGCGTCTATTTAGTGGAGCCGTACCTATCTTCTTTAGTCCGGTGAATTCGCCCCT
mTcrb_GTAC_3_AP2	AAGTTCTTTCCCGATGTCATCTGATTCCTTTGACTCACATTTTTGCGTCTATTTAGTGGAGCCGTACCTATCTTCTTTCATACCTTTGTCTCCTTGAA
mTcrb_GTAC_4_AP3	TTTCATCCTCACTGCCATAATCTACGAGTTTGCAGTCACGTTTTGCGTCTATTTAGTGGAGCCGTACCTATCTTCTTTGGTACAGCAAGTCAGCTGGA
mIl6_CGAT_3_AP1	CTGGAGCCCACCAAGAACGATCTACGATTTTACCAGTGGCTTTTGCGTCTATTTAGTGGAGCCCGATCTATCTTCTTTTGTCTGTAGCTCATTCTGCT
mIl6_CGAT_4_AP2	CTGGAGTACATGAAGAACAACTGATTCCTTTGACTCACATTTTTGCGTCTATTTAGTGGAGCCCGATCTATCTTCTTTTTCTGGAGTACCATAGCTAC
mIl6_CGAT_5_AP3	GTTTCTTGGAATGTATAAGTTCTACGAGTTTGCAGTCACGTTTTGCGTCTATTTAGTGGAGCCCGATCTATCTTCTTTTTCTGTTACCTAGCCAGATG
mIl12_CAGT_3_AP1	CTCTTTTTGGCCACCCTTGCTCTACGATTTTACCAGTGGCTTTTGCGTCTATTTAGTGGAGCCCAGTCTATCTTCTTTTGTGTCAATCACGCTACCTC
mIl12_CAGT_4_AP2	CAGCAGATCATTCTAGACAACTGATTCCTTTGACTCACATTTTTGCGTCTATTTAGTGGAGCCCAGTCTATCTTCTTTCACTTCAGAATCACAACCAT
mIl12_CAGT_5_AP3	TCCCCTCCATCGCTTCTCTCTCTACGAGTTTGCAGTCACGTTTTGCGTCTATTTAGTGGAGCCCAGTCTATCTTCTTTGCACAGCTACCTCAGCATGG
mCd68_AGCT_3_AP1	TCCTCTTCCAAGAGAAGGCATCTACGATTTTACCAGTGGCTTTTGCGTCTATTTAGTGGAGCCAGCTCTATCTTCTTTGGGAAGTGAGGCTTTTCATT
mCd68_AGCT_4_AP2	CAAATTCAAATCCGAATCCTCTGATTCCTTTGACTCACATTTTTGCGTCTATTTAGTGGAGCCAGCTCTATCTTCTTTCTTGTGTTCAGCTCCAAGCC
mCd68_AGCT_5_AP3	AGCATCTGCCCCAGTCCACTTCTACGAGTTTGCAGTCACGTTTTGCGTCTATTTAGTGGAGCCAGCTCTATCTTCTTTTCAACCTACCAGCCCCTCTG
mTnf_CCAA_4_AP1	GCCAGGAGGGAGAACAGAAATCTACGATTTTACCAGTGGCTTTTGCGTCTATTTAGTGGAGCCCCAACTATCTTCTTTGCGAGGACAGCAAG

	GGACTA
mTnf_CCAA_5_AP2	GTCTACTCCCAGGTTCTCTTCTGATTCCTTTGACTCACATTTTTGCGTCTATTTAGTGGAGCCCCAACTATCTTCTTTCAGCCGATGGGTTGTACCTT
mTnf_CCAA_6_AP3	CTACTCAGAAACACAAGATGTCTACGAGTTTGCAGTCACGTTTTGCGTCTATTTAGTGGAGCCCCAACTATCTTCTTTGTGCTCAGAGCTTTCAACAA
mCd40L_CGTA_1_AP1	CCTAAGAACTTACAATCTGATCTACGATTTTACCAGTGGCTTTTGCGTCTATTTAGTGGAGCCCGTACTATCTTCTTTAATCCCAAGGGACCCTGCTC
mCd40L_CGTA_2_AP2	TGTGTTTGTCAACGTGACTGCTGATTCCTTTGACTCACATTTTTGCGTCTATTTAGTGGAGCCCGTACTATCTTCTTTGAATTACAAGCTGGTGCTTC
mCd40L_CGTA_3_AP3	CTATTATGTCTACACTCAAGTCTACGAGTTTGCAGTCACGTTTTGCGTCTATTTAGTGGAGCCCGTACTATCTTCTTTACGGTTAAAAGAGAAGGACT
mCd69_CTAG_1_AP1	AAGGAAGAAAACAAAAGCACTCTACGATTTTACCAGTGGCTTTTGCGTCTATTTAGTGGAGCCCTAGCTATCTTCTTTAAACCTCTGTAGCGTATTTC
mCd69_CTAG_2_AP2	CTGCTAATCACATCAAGGATCTGATTCCTTTGACTCACATTTTTGCGTCTATTTAGTGGAGCCCTAGCTATCTTCTTTCTGAAACTGTCACCAACTGA
mCd69_CTAG_3_AP3	AAATGAAGCTAATCAGACATTCTACGAGTTTGCAGTCACGTTTTGCGTCTATTTAGTGGAGCCCTAGCTATCTTCTTTGAACATTGGATTGGGCTGAA
mSCGB1A1_GGCC_AP4	CTCACTGGATTTCAGAGATATGCTTGTGGTAGCAAATATTTTGCGTCTATTTAGTGGAGCCGGCCCTATCTTCTTTAAGCAAGATTTAAGATTCTGAAG
mSftpc_CGCG_AP4	ATTCTTCCGGGAGAGACCTGTGCTTGTGGTAGCAAATATTTTGCGTCTATTTAGTGGAGCCCGCGCTATCTTCTTTTGATACTGGTTCCGAGTCCG
mCD86_CTGA_ap1	TGCAGACTCTTAAGATCTGGTCTACGATTTTACCAGTGGCTTTTGCGTCTATTTAGTGGAGCCCTGACTATCTTCTTTGTCCATCTTGATTCACCCTG
mCD86_CTGA_ap2	GGATGGGAGTGGTATATTTGCTGATTCCTTTGACTCACATTTTTGCGTCTATTTAGTGGAGCCCTGACTATCTTCTTTGACAGCTACCTCTTCAGTCA
mCD86_CTGA_ap3	TCAGTGATCGCCAACTTCAGTCTACGAGTTTGCAGTCACGTTTTGCGTCTATTTAGTGGAGCCCTGACTATCTTCTTTAACAGACATTAACAGAACTG
mElane_GATC_ap1	CATTATCCGAAGCCATAATGTCTACGATTTTACCAGTGGCTTTTGCGTCTATTTAGTGGAGCCGATCCTATCTTCTTTTTTGCAGATTGGATCAATTC
mElane_GATC_ap2	GCCAACGTGCAGGTGGCCCACTGATTCCTTTGACTCACATTTTTGCGTCTATTTAGTGGAGCCGATCCTATCTTCTTTATGGCTCCGCTACCATTAAC
mElane_GATC_ap3	CTATCCAGCCGGACTCTGGCTCTACGAGTTTGCAGTCACGTTTTGCGTCTATTTAGTGGAGCCGATCCTATCTTCTTTCCACCATGGCCCTTGGCAGA
mIl12b_GCTA_ap1	AGGACACAATGGCCACACTCTCTACGATTTTACCAGTGGCTTTTGCGTCTATTTAGTGGAGCCGCTACTATCTTCTTTGAAGATACTACGGCTACCTC

mIl12b_GCTA_ap2	CATTAGCTTTTGTGACAACCCTGATTCCTTTGACTCACATTTTTGCGTCTATTTAGTGGAGCCGCTACTATCTTCTTTCAATCAGGGCTGCGTAGGTA
mIl12b_GCTA_ap3	ACTCCGGACGGTTCACGTGCTCTACGAGTTTGCAGTCACGTTTTGCGTCTATTTAGTGGAGCCGCTACTATCTTCTTTGAAGTGTGAAGCACCAAATT
mTbx21_GTCA_ap1	TTCTTCTGTTTTGCATCACTTCTACGATTTTACCAGTGGCTTTTGCGTCTATTTAGTGGAGCCGTCACTATCTTCTTTAGAGTGGTGTCTGGATGTAT
mTbx21_GTCA_ap2	GGATCCTCAGAGGAACAGGGCTGATTCCTTTGACTCACATTTTTGCGTCTATTTAGTGGAGCCGTCACTATCTTCTTTTGCCCATGGACCCGGGCCTG
mTbx21_GTCA_ap3	CCGGGCGCACAGGACCCGACTCTACGAGTTTGCAGTCACGTTTTGCGTCTATTTAGTGGAGCCGTCACTATCTTCTTTATCGTTTCTTCTATCCCGAG
mCd4_AGTC_5_AP1	AGTCCACCAAAGACCTCAAGTCTACGATTTTACCAGTGGCTTTTGCGTCTATTTAGTGGAGCCAGTCCTATCTTCTTTCAAAGAGGTGTCCGTACAAA
mCd4_AGTC_6_AP2	CCAGCCCTGGATCTCCTTCTCTGATTCCTTTGACTCACATTTTTGCGTCTATTTAGTGGAGCCAGTCCTATCTTCTTTGCAGAGAAGGATTCTTTCTT
mCd4_AGTC_7_AP3	CATGTCCAACCTAAGGGTTCTCTACGAGTTTGCAGTCACGTTTTGCGTCTATTTAGTGGAGCCAGTCCTATCTTCTTTAGTGGTTCCAAAGTTCTCTC

Supplementary Table 4

Sequencing-by-ligation oligonucleotides

name	sequence	5' modification	3' modification
Anchor oligo B2_DO_27_U_5AF750	/5Alex750N/UGCGUCUAU UUAGUGGAGCC	Alexa Fluor 750	-
Cy5_0A_4N	ANNNCTATC	Phosphorylation	Cy5
Cy3_0G_4N	GNNNCTATC	Phosphorylation	Cy3
TR_0C_4N	CNNNCTATC	Phosphorylation	Texas Red
AF488_0T_4N	TNNNCTATC	Phosphorylation	Alexa Fluor 488
Cy5_1A_4N	NANNCTATC	Phosphorylation	Cy5
Cy3_1G_4N	NGNNCTATC	Phosphorylation	Cy3
TR_1C_4N	NCNNCTATC	Phosphorylation	Texas Red
AF488_1T_4N	NTNNCTATC	Phosphorylation	Alexa Fluor 488
Cy5_2A_4N	NNANCTATC	Phosphorylation	Cy5
Cy3_2G_4N	NNGNCTATC	Phosphorylation	Cy3
TR_2C_4N	NNCNCTATC	Phosphorylation	Texas Red
AF488_2T_4N	NNTNCTATC	Phosphorylation	Alexa Fluor 488
Cy5_3A_4N	NNNACTATC	Phosphorylation	Cy5
Cy3_3G_4N	NNNGCTATC	Phosphorylation	Cy3
TR_3C_4N	NNNCCTATC	Phosphorylation	Texas Red
AF488_3T_4N	NNNTCTATC	Phosphorylation	Alexa Fluor 488
