# Supplementary material for: Spatial and temporal localization of immune transcripts defines hallmarks and diversity in the tuberculosis granuloma
Source: Nat Commun. 2019 Apr 23;10:1823. doi: 10.1038/s41467-019-09816-4 (PMC6479067; doi:10.1038/s41467-019-09816-4)
Supplement: Supplementary file 3 — Reporting Summary [file 41467_2019_9816_MOESM3_ESM.pdf]

nature research

Corresponding author(s): Martin Rottenberg

Last updated by author(s): Mar 15, 2019

Reporting Summary

Nature Research wishes to improve the reproducibility of the work that we publish. This form provides structure for consistency and transparency in reporting. For further information on Nature Research policies, see Authors & Referees and the Editorial Policy Checklist.

Statistics

For all statistical analyses, confirm that the following items are present in the figure legend, table legend, main text, or Methods section.

n/a	Confirmed	
☐	☒	The exact sample size (*n*) for each experimental group/condition, given as a discrete number and unit of measurement
☐	☒	A statement on whether measurements were taken from distinct samples or whether the same sample was measured repeatedly
☐	☒	The statistical test(s) used AND whether they are one- or two-sided<br>*Only common tests should be described solely by name; describe more complex techniques in the Methods section.*
☒	☐	A description of all covariates tested
☐	☒	A description of any assumptions or corrections, such as tests of normality and adjustment for multiple comparisons
☐	☒	A full description of the statistical parameters including central tendency (e.g. means) or other basic estimates (e.g. regression coefficient) AND variation (e.g. standard deviation) or associated estimates of uncertainty (e.g. confidence intervals)
☒	☐	For null hypothesis testing, the test statistic (e.g. *F*, *t*, *r*) with confidence intervals, effect sizes, degrees of freedom and *P* value noted<br>*Give P values as exact values whenever suitable.*
☒	☐	For Bayesian analysis, information on the choice of priors and Markov chain Monte Carlo settings
☒	☐	For hierarchical and complex designs, identification of the appropriate level for tests and full reporting of outcomes
☐	☒	Estimates of effect sizes (e.g. Cohen's *d*, Pearson's *r*), indicating how they were calculated

Our web collection on statistics for biologists contains articles on many of the points above.

Software and code

Policy information about availability of computer code

Data collection	Images were aquired using Zen Imaging Software. Processing of sequencing images to positional data was performed using Matlab (2017b) and CellProfiler scripts (v.2.1.1). All scripts and a description can be found in our repository under DOI: https://doi.org/10.6084/m9.figshare.7663010
Data analysis	For downstream analysis, we used customized Matlab scripts (described under DOI: https://doi.org/10.6084/m9.figshare.7663010), for multivariant analysis ClustVis (https://biit.cs.ut.ee/clustvis/), and for network analysis the 2 applications InsituNet and DynetAnalyzer on the open software Cytoscape platform (http://apps.cytoscape.org).

For manuscripts utilizing custom algorithms or software that are central to the research but not yet described in published literature, software must be made available to editors/reviewers. We strongly encourage code deposition in a community repository (e.g. GitHub). See the Nature Research guidelines for submitting code & software for further information.

Data

Policy information about availability of data

All manuscripts must include a data availability statement. This statement should provide the following information, where applicable:

- Accession codes, unique identifiers, or web links for publicly available datasets
- A list of figures that have associated raw data
- A description of any restrictions on data availability

All raw images are available on request, all positional csv files, DAPI images for plotting and high resolution H&E staining of in situ-sequenced lungs can be found under DOI: https://doi.org/10.6084/m9.figshare.7663010. All scripts for the in situ sequencing processing pipeline and downstream analysis can be found under the same DOI. All figures are associated with the raw data included. The data are publicly available without restrictions.
The source data underlying figures 1B, 2C, 3D, 4E, 5A-E, 7 D-I and supplementary figures 2E and 5C are provided as a source data file.

Field-specific reporting

Please select the one below that is the best fit for your research. If you are not sure, read the appropriate sections before making your selection.

☒ Life sciences ☐ Behavioural & social sciences ☐ Ecological, evolutionary & environmental sciences

For a reference copy of the document with all sections, see nature.com/documents/nr-reporting-summary-flat.pdf

Life sciences study design

All studies must disclose on these points even when the disclosure is negative.

Sample size	As indicate in the results sections a limitation of our study is the small number of independent sections examined. This is due to the extensive image acquisition and data processing. Statistics were performed by comparing frequencies of transcripts in different areas of the lung or in different regions of the granuloma but also by comparison of determinations in consecutive sections, which confirmed the specificity of the signals. Three consecutive sections were analyzed per condition. The results presented were confirmed in one independent sample for each condition.
Data exclusions	If the mRNA species was undetectable in more than two areas in an analysed section, it was excluded from analysis.
Replication	We have analysed three consecutive sections for each sample to verify the reproducibility of our data.
Randomization	Not relevant
Blinding	Not relevant

Reporting for specific materials, systems and methods

We require information from authors about some types of materials, experimental systems and methods used in many studies. Here, indicate whether each material, system or method listed is relevant to your study. If you are not sure if a list item applies to your research, read the appropriate section before selecting a response.

Materials & experimental systems

n/a	Involved in the study	
☐	☒	Antibodies
☒	☐	Eukaryotic cell lines
☒	☐	Palaeontology
☐	☒	Animals and other organisms
☒	☐	Human research participants
☒	☐	Clinical data

Methods

n/a	Involved in the study	
☒	☐	ChIP-seq
☒	☐	Flow cytometry
☒	☐	MRI-based neuroimaging

Antibodies

Antibodies used

1. rat anti-mouse CD45R/B220 antibody (clone RA3-6B2, BD Pharmingen, catalog #550286)
2. rat anti-mouse CD3 (clone CD3-12, abcam, catalog # ab11089)
3. rabbit polyclonal anti-mouse CD68 (abcam, catalog # ab125212)

Validation

1. developed for immunohistochemistry,reacts with Mouse, selected references from manufacturer's webpage (mouse):
- Asensi V, Kimeno K, Kawamura I, Sakumoto M, Nomoto K. Treatment of autoimmune MRL/lpr mice with anti-B220 monoclonal antibody reduces the level of anti-DNA antibodies and lymphadenopathies. Immunology. 1989; 68(2):204-208.
- Coffman RL. Surface antigen expression and immunoglobulin gene rearrangement during mouse pre-B cell development. Immunol Rev. 1982; 69:5-23.
2. developed for immunohistochemistry, reacts with Mouse, Horse, Chicken, Dog, Human, Pig, Rhesus monkey, selected references from manufacturer's webpage (mouse):
- Wang Q et al. Vascular niche IL-6 induces alternative macrophage activation in glioblastoma through HIF-2a. Nat Commun 9:559 (2018). IHC-P ; Mouse
- Takeuchi Y et al. Development of Novel Mouse Model of Ulcers Induced by Implantation of Magnets. Sci Rep 7:4843 (2017). IHC-P ; Mouse .
3. developed for immunohistochemistry, reacts with Mouse, Rat, Human, selected references from manufacturer's webpage:
- Toullec A et al. HIF-1a Deletion in the Endothelium, but Not in the Epithelium, Protects From Radiation-Induced Enteritis. Cell Mol Gastroenterol Hepatol 5:15-30 (2018). IHC-P ; Mouse .
- Clarke JR et al. Alzheimer-associated Aß oligomers impact the central nervous system to induce peripheral metabolic deregulation. EMBO Mol Med 7:190-210 (2015). IHC (PFA fixed) ; Mouse .

Animals and other organisms

Policy information about studies involving animals; ARRIVE guidelines recommended for reporting animal research

Laboratory animals	C57BL/6 mice were purchased from Janvier labs, C3HeB/FeJ mice were received from Igor Kramnik (BU, Boston, MA).
Wild animals	This study did not involve wild animals
Field-collected samples	This study did not involve samples collected from the field.
Ethics oversight	Stockholm North Animal Research Research Ethical Committee

Note that full information on the approval of the study protocol must also be provided in the manuscript.
